# Comparison of Colorectal Cancer Surgery Services During COVID-19 First Wave With Pre-COVID Time

**DOI:** 10.7759/cureus.17585

**Published:** 2021-08-30

**Authors:** Muhammad Umair Rashid, Syed Soulat Raza, Pradeep Thomas, Stelios Vakis

**Affiliations:** 1 General Surgery, University Hospitals of Derby and Burton NHS Trust, Staffordshire, GBR; 2 Surgery, University Hospitals of Birmingham NHS Trust, Birmingham, GBR; 3 General and Colorectal Surgery, University Hospitals of Derby and Burton NHS Trust, Staffordshire, GBR

**Keywords:** colorectal surgery, lower gi surgery, colorectal cancer, open and laparoscopic surgery, covid 19, colorectal surgeon

## Abstract

Introduction

The first confirmed case of COVID-19 in the United Kingdom (UK) was reported on 29 January 2020. The country saw the peak of infection between March and May of 2020. The result was a change in the practice of how we treat most surgical conditions including cancer. We continued providing service to our colorectal cancer patients at a District General Hospital. The aim of this study was to compare our provision of colorectal cancer service during the peak of the pandemic to that of the pre-COVID time in our hospital.

Methods

We collected data of all colorectal cancer patients who underwent surgery between 1 March 2020 and 30 April 2020 in our hospital. The comparative data were collected for similar patients during the same time frame in 2019. A detailed data set was compiled on Microsoft Excel (Microsoft Corp, Washington) and analysed using IBM SPSS Statistics for Windows, Version 21.0 (Released 2012. IBM Corp, Armonk, NY).

Results

The two groups were comparable in demographics including age, BMI, gender, and Charlson comorbidity index. Time from decision- to-treat to surgery, post-operative HDU/ITU stay, and overall length of stay was shorter in the COVID group than the Pre-COVID group without any significant statistical difference. There was no statistically significant difference between the two groups in Calvien-Dindo complications grade 1 and 2. No mortality was reported due to direct or indirect consequences of COVID-19 infection. More open procedures were performed in our department during the first wave of COVID-19 in the UK compared to Pre-COVID time.

Conclusions

Despite the challenges we faced during the peak of the COVID-19 pandemic, we managed to provide standard care to our colorectal cancer patients with comparable post-operative surgical and oncological outcomes.

## Introduction

The first case of COVID-19 in the United Kingdom (UK) was reported on 29 January 2020. On 3 March 2020, the Government of UK published an action plan to tackle coronavirus. The plan stated that the health and social care system will be prepared to respond to all eventualities of a potential future pandemic, and this might mean that other services are reduced temporarily [1]. Subsequently National Health Service (NHS) England published guidance to management of non-COVID patients requiring urgent acute treatment such as cancer. The guidelines suggested prioritizing the patients with priority level 1 given to patients requiring curative therapy with a high (>50%) chance of success [2]. Most colorectal cancer patients fall into this category. Association of Coloproctology in Great Britain and Ireland (ACPGBI) published its consideration for multidisciplinary management of patients with colorectal cancer during the COVID-19 pandemic. It was recommended that consideration be given to the likelihood of patients requiring access to planned or unplanned critical care, planning strategies to mitigate the risk of needing critical care, and considering current and predicted critical care capacity during the multidisciplinary team meetings. In the same month, Intercollegiate general surgery guidelines on COVID-19 were published. This advocated risk-reducing strategies including the formation of stoma and avoidance of laparoscopy. This had a clear impact on the provision of service to colorectal cancer patients. However, on 30 March 2020, the European Society of Coloproctology (ESCP) endorsed the European Association of Endoscopic Surgeons (EAES) and the Society of American Gastrointestinal and Endoscopic Surgeons (SAGES) for the management of colorectal cancer patients during the COVID-19 pandemic. The recommendations suggested that there is little evidence regarding the relative risks of minimally invasive surgery (MIS) versus the conventional open approach, specific to COVID-19. Hence, a change of practice is not needed until stronger evidence comes forth [3]. In addition to that, there was guidance from the Royal College of Radiologists regarding pre-operative CT chest for all patients undergoing elective colorectal surgery, which was subsequently rolled back [4]. In short, the guidance on management of colorectal patients during the first peak of the pandemic was slightly conflicting and changed frequently in those unprecedented times. There was no change in the two weeks wait referrals system and endoscopic examination was performed for patients with suspected cancer referral during the pandemic. The aim of our study was to analyse our provision of care to colorectal cancer patients and compare the surgical and oncological outcomes with the patients from the pre-COVID era.

## Materials and methods

We identified all patients undergoing elective surgery for colorectal cancer between 1 March 2020 and 30 April 2020 in Queens Hospital Burton, University Hospitals of Derby and Burton NHS Trust. From the departmental registry, 22 patients were identified for this period. Ten patients who underwent colorectal resection during the same time frame a year ago, from 1 March 2019 to 30 April 2019, were identified. Data collected from the registry were categorized into two groups: COVID and Pre-COVID. Basic demographics including age, BMI, gender, American Society of Anesthesiology (ASA) score, Charlson comorbidity index, presentation of the patient (asymptomatic vs symptomatic) were collected. Pre-operative and post-operative quality indicators such as the duration from the multidisciplinary team (MDT) decision to operate to the date of surgery, duration of high dependency unit (HDU) stay, intensive treatment unit (ITU) stay, and overall hospital stay was included in the data. Oncological data included histological type, location of tumour, and clearance of resection margins. Surgical data of type of approach (laparoscopic vs open), Clavien-Dindo complications, and inpatient or 30-day mortality were added to the data. Continuous data were presented as mean ± SD. As the data was not normally distributed, continuous variables were compared between the two groups using the Mann-Whitney U test (p<0.05). Fisher’s exact test was used to compare the association between categorical variables in the two groups. Analysis was performed using IBM SPSS Statistics for Windows, Version 21.0 (Released 2012. IBM Corp, Armonk, NY).

## Results

A total of 32 colorectal cancer patients underwent surgery from our department in the time period used for the study; 22 during the COVID period and 10 from the Pre-COVID era. The baseline demographic characteristics of patients were comparable between the two groups (Table 1). The mean +/- S.D age was 74.2 ± 7 years vs. 69.2 ± 13 years in COVID and Pre-COVID era respectively. Similarly, BMI for COVID and Pre-COVID era was 29±5 and 27±4 respectively. The two groups were comparable on gender distribution and Charlson comorbidity index with a mean index of group and 6.1±1.79 in Pre-COVID era group and 5.68±1.6 in COVID era. In the pre-COVID group, 80% (eight) patients presented with symptoms as opposed to only 45% (10) patients in the COVID group but this was not statistically significant.

**Table 1:** Baseline demographics in both groups (n=32) p-value calculated by Man Whitney U test, statistical significance taken at <0.5 *Denotes numbers (percentages), rest of the results expressed as means ±SD SD: standard deviation

Variables		Pre-COVID (n=10)	COVID (n=22)	p-Value
Age (years)				0.589
	Mean ± SD	69 ± 13	74 ± 7	
Gender (*)				0.595
	Male	7(70%)	16(73%)	
	Female	3(30%)	6(27%)	
Body Mass Index				0.411
	Mean ± SD	27 ± 4	29 ± 5	
ASA Grade				0.54
	Mean ± SD	2.50 ±0.52	2.91±0.61	
Charlson Comorbidity Index				0.65
	Mean ± SD	6.10± 1.79	5.68±1.56	
Charlson Comorbidity Index (*)				0.446
	≤ 5	3(30%)	11(50%)	
	˃ 5	7(70%)	11(50%)	
Presentation of Cancer (*)				1.00
	Asymptomatic	2(20%)	12(55%)	
	Symptomatic	8(80%)	10(45%)	

In terms of pre- and post-operative quality indicators, both groups had similar duration from MDT to surgery 23±9 and 29±5 days in pre and post COVID groups (Table 2). There was no statistically significant difference in time from the first referral to MDT discussion. However, the time from initial referral to surgery varied because of rectal cancer patients receiving neoadjuvant chemotherapy. Hence, this was not compared between the two groups. The mean length of post-operative stay was slightly shorter, 5±2 days in COVID era patients, compared with 8±9 days in the Pre-COVID group (p= 0.589). The mean stay in HDU/ITU was 1±3 days in Pre-COVID patients compared to 0.1±0.5 days, with only two patients requiring HDU stay post-operatively, in the COVID group (p= 0.366). Overall, there was a trend to shorter post-operative HDU/ITU and overall hospital stays during COVID but these were not significant.

**Table 2:** Pre- and post-operative quality indicators for both groups (n=32) p-value calculated by Man Whitney U test, statistical significance taken at <0.5 Results are expressed as Mean ± SD SD: standard deviation; MDT: multidisciplinary team; HDU: high dependency units; ITU: intensive treatment unit

Variables	Pre-COVID	COVID	p-Value
Duration from MDT to Surgery (Days)	23 ± 9	29 ± 5	0.325
Length of Stay (Days)	8 ± 9	5 ± 2	0.589
Post-operative HDU/ ITU stay (Days)	1 ± 3	0.1±0.5	0.366

There was no difference in the chemotherapy regime used in both groups of patients. The oncological variables between the two groups were also similar. These are detailed in Table 3 along with surgical outcomes. Four patients in the COVID group had Clavien-Dindo grade 2 post-operative complications. Out of these, two were chest infections and both were negative for COVID-19 infection upon testing. In the pre-COVID era, two patients had Calvien-Dindo grade 3b post-operative complications with a return to theatre due to bleeding and bowel obstruction. There was no statistically significant difference between the two groups in post-operative complications (Table3). None of the patients had an anastomotic leak. The only clear difference we found was significantly more open procedures performed during the COVID era (68%) compared to the Pre-COVID era (20%) (p-value 0.021). Conversion to open surgery was in three (30%) patients in the Pre-COVID group compared to no conversion in the COVID group. Four (18%) patients in the COVID era had an end colostomy compared to none in the Pre-COVID era. Patients in both groups did not have in-hospital or 30-day mortality or a re-admission.

**Table 3:** Oncological and surgical data for both groups (n=32) p-value calculated by Fishers Exact test, statistical significance taken at <0.5

Variables		Pre-COVID (n=10)	COVID (n=22)	p-Value
Location of Tumor				0.476
	Colonic	7 (70%)	12 (55%)	
	Rectal	3 (30%)	10 (45%)	
Histology of Tumor				0.534
	Adenocarcinoma	9 (90%)	21 (95%)	
	Mucinous	1 (10%)	1(5%)	
Resection margin on histology				0.224
	No Residual tumour (R0)	8 (80%)	21(95%)	
	Residual tumour (R1)	2 (20%)	1(5%)	
Nature of the Procedure				0.021
	Laparoscopic surgery	8 (80%)	7 (32%)	
	Open surgery	2 (20%)	15 (68%)	
Clavein-Dindo Complications				1.000
	Grade 1	7 (70%)	18 (72%)	
	Grade 2	1 (10%)	4 (80%)	
	Grade 3	2 (20%)	0	

## Discussion

The outbreak of COVID-19 posed major healthcare, scientific, and economic challenges globally [5]. These ranged from shortage of personal protective equipment and testing kits to lack of clear guidelines on the management of non-COVID patients during the pandemic. Most of the recommendations during the first peak of pandemic in UK were based on expert opinions [6].

Compounding these factors, the surgeons faced the additional dilemma on whom to operate and which services to cut back. Elective procedures such as hernia repair, laparoscopic cholecystectomy, and fundoplication were relatively straightforward to put on hold. Keeping the limited ITU/HDU resources in mind, we had to further evaluate cancer patients to choose patients who will receive maximum benefit with minimal ITU/HDU support. This required a thorough consideration and assessment on a case-to-case basis along with re-prioritizing of current waiting lists.

Early experience from China suggested that the COVID-19 pandemic clearly influenced the care of patients with colorectal diseases; elective surgeries were especially more affected than emergency surgeries [7]. Utilizing our newly designed list prioritization and careful patient selection, we were able to increase our colorectal resection numbers to almost double when compared to the same time frame a year ago.

In our experience, no major difference was observed in management of colorectal patients in comparison with the Pre-COVID era. Most of the surgeons in China reported colorectal cancer was the main indication for colorectal surgery during the COVID-19 pandemic. This was also true in our practice as all benign elective surgeries were postponed during the pandemic and only elective cancer and emergency surgery was continued. Surgeons were also concerned about a compromise of the care of their cancer patients from the diversion of health care resources [8]. This was a concern in our team as well but our results show that there was no compromise in provision of standard of care to colorectal patients when compared with pre-COVID era.

One of the earlier studies indicated that patients with cancer had worse outcomes from COVID-19 [9]. The study also reported the median age of these patients was significantly higher than for those without cancer, suggesting that older age was associated with worse COVID-19 outcomes. Other studies concurred these results showing that many of the more seriously affected patients with COVID-19 were elderly. Same is true for cancer as it is predominately a disease of the elderly [10]. However, the average age of our patients was 74.2 ± 7 years, which was higher than the average age of patients in pre-COVID era. There was a real risk of asymptomatic COVID-19 infection in patients and its associated risk with surgery, but when weighted against the risk from delayed cancer treatment or no treatment and considering cancer-related morbidity and mortality, the decisions became simpler. These risks and benefits were discussed explicitly with patients during consent procedure. Our results show no transmission of COVID-19 during peri-operative period. Our results also show a higher threshold for patients requiring HDU/ITU stay during the pandemic. However, two patients required re-operation for Clavien-Dindo 3b complications resulting in unplanned HDU/ITU stay in the pre-COVID group, which needs to be accounted for increased stay in HDU/ITU. The overall length of stay was shorter in the COVID group even with more patients having an open surgery rather than laparoscopic approach. This may reflect a more careful patient selection and patient psychology the during pandemic to avoid longer in hospital stays.

## Conclusions

We accept that there are several limitations in our study including the retrospective nature and the small sample size of the cohort, but in conclusion, we did not find any difference in our practice. In fact, if anything. our resection numbers increased and we focused on the provision of urgent and emergency services with whatever limited resources we had available at the time. We all acknowledge that the COVID-19 pandemic is far from reaching a resolution; therefore, it is vital that we as a surgical community share our experiences and results to enable us to be better prepared for the future.
